# 1724. Murine Efficacy Studies of Sulopenem Against *Bacillus anthracis*

**DOI:** 10.1093/ofid/ofac492.1354

**Published:** 2022-12-15

**Authors:** Sailaja Puttagunta, Steven I Aronin, Michael Dunne, Stephanie A Halasohoris, Ashley Babyak, Mary K Hourihan, James M Meinig

**Affiliations:** Iterum Therapeutics, Old Saybrook, Connecticut; Iterum Therapeutics, Old Saybrook, Connecticut; Bill & Melinda Gates Medical Research Institute, Old Saybrook, Connecticut; US Army Medical Research Institute of Infection Diseases, Fort Detrick, Maryland; US Army Medical Research Institute of Infection Diseases, Fort Detrick, Maryland; US Army Medical Research Institute of Infection Diseases, Fort Detrick, Maryland; US Army Medical Research Institute of Infection Diseases, Fort Detrick, Maryland

## Abstract

**Background:**

Sulopenem is a thiopenem β-lactam antibiotic being developed for the treatment of infections caused by multi-drug resistant bacteria. Sulopenem is available as intravenous and oral pro-drug formulations, and its activity aligns with the most urgent drug-resistant antimicrobial threats defined by the CDC. Sulopenem possesses potent activity against species of the Enterobacterales that encode ESBLs or AmpC-type β-lactamases that confer resistance to third generation cephalosporins. It has also demonstrated good *in vitro* microbiological activity against a range of bacterial pathogens including penicillin resistant *S. pneumoniae,* β-lactamase-producing *H. influenza,* and *M. catarrhalis*. Sulopenem is also active *in vitro* against a number of bio-threat pathogens at concentrations likely to be achieved after oral dosing in humans and meets criteria to be tested in the murine models of *Bacillus anthracis, Yersinia pestis, Burkholderia mallei,* and *Burkholderia pseudomallei*. The development of novel medical countermeasures (MCMs) is critical to biodefense preparedness for both military and public health.

**Methods:**

Female BALB/c mice were challenged with *B. anthracis* Ames spores by whole-body aerosol, with an average challenge of 15 x LD_50_ and randomly divided into cohorts of 10 mice per group. At 24h post-exposure prophylaxis (PEP), which represents therapy before onset of clinical symptoms, mice were treated q8h for 14 days with vehicle (saline, IP), ciprofloxacin (30mg/kg, IP) or sulopenem etzadroxil (50, 25, or 12.5 mg/kg, PO). Mice were monitored for a total of 30 days and data analyzed to determine the effects of sulopenem etzadroxil on survival as compared to the positive treatment control, ciprofloxacin, using Log-Ranks tests for the pair wise comparisons with SAS software.

**Results:**

**Figure 1:**
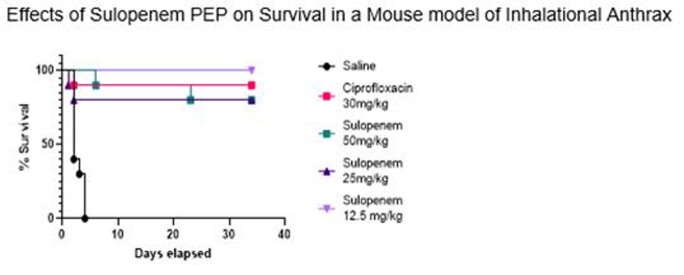

**Conclusion:**

Sulopenem is active *in vivo* in the murine model of *B. anthracis.* Survival in the sulopenem treated groups was not statistically inferior to the ciprofloxacin positive control, a standard-of-care for PEP of *B. anthracis*. These results support further development of sulopenem for treating *B. anthracis* as a novel broad-spectrum and orally available MCM.

**Disclosures:**

**Sailaja Puttagunta, MD**, Iterum Therapeutics: Stocks/Bonds **Steven I. Aronin, MD**, Iterum Therapeutics: Stocks/Bonds **Michael Dunne, MD**, Iterum Therapeutics: Advisor/Consultant|Iterum Therapeutics: Board Member|Iterum Therapeutics: Stocks/Bonds.

